# Transthyretin and Vitamin A Metabolism: A Review for the Cardiac Amyloidosis Specialist

**DOI:** 10.3390/jcdd13050205

**Published:** 2026-05-12

**Authors:** Donclair Brown, Vishakha Modak, Aladin Altic, Ali Al Zuwayny, James Tauras

**Affiliations:** 1 Montefiore Medical Center, Albert Einstein College of Medicine, Bronx, NY 10467, USA; jtauras@montefiore.org; 2 Jacobi Medical Center, Albert Einstein College of Medicine, NYC Health and Hospitals, Bronx, NY 10461, USA; modakv@nychhc.org; 3 Jacobi Medical Center/North Central Bronx, Albert Einstein College of Medicine, NYC Health and Hospitals, Bronx, NY 10467, USA; 4 Richmond University Medical Center, Staten Island, NY 10310, USA; aalzuwayny@rumcsi.org

**Keywords:** vitamin A, ATTR cardiomyopathy, transthyretin, cardiac amyloid

## Abstract

Transthyretin (TTR) amyloidosis is a systemic, progressive, and fatal disease. TTR is integral in vitamin A (retinol) transport via its binding to retinol binding protein 4 (RBP4). Current and emerging therapies for TTR amyloid cardiomyopathy (ATTR-CM), including RNAi therapies and potentially CRISPR-based therapies, reduce hepatic transthyretin production and hence decrease serum RBP4, which decreases circulating vitamin A levels. However, despite these reductions in circulating vitamin A, hepatic reserves and alternative delivery mechanisms may prevent clinical manifestations of vitamin A deficiency. Vitamin A functions as a key regulator of immunity, antioxidant function, cell growth and differentiation and vision. This paper aims to serve as a comprehensive review of vitamin A and its metabolites, their transport, and their function in human health and disease. Additionally, we seek to synthesize the relevant outcomes and safety data of TTR silencing therapies and how they relate to circulating vitamin A levels and vitamin A-related clinical outcomes in a manner that is relevant to the cardiac amyloidosis specialist.

## 1. Introduction

Transthyretin (TTR) amyloidosis is a systemic, progressive, and fatal disease. The development of noninvasive diagnostic pathways and effective treatments for transthyretin cardiomyopathy (ATTR-CM) has allowed for increased and earlier detection of the disease. TTR itself is a homotetramer with many roles in human homeostasis, including but not limited to thyroid hormone transport and vitamin A transport via retinol binding protein 4 (RBP4). Some of the current and emerging therapies for ATTR-CM, including RNAi and CRISPR therapies, significantly reduce circulating transthyretin and hence RBP4, thereby playing a role in vitamin A transportation and metabolism. Notably, reductions in circulating vitamin A do not necessarily cause clinical manifestations of deficiency, as compensatory mechanisms, such as preserved hepatic vitamin A stores and alternative retinol transport pathways, may help maintain vitamin A homeostasis during a TTR-deficient state. The role of vitamin A supplementation, the amount of supplementation needed, and the potential risks faced by ATTR-CM patients on long-term TTR-silencing therapies are poorly understood and a cause of disagreement amongst amyloidosis specialists. This review outlines the role of TTR and TTR-silencing therapies on retinol-binding protein and vitamin A metabolism to help facilitate a greater understanding of these critical issues in the care of the cardiac amyloidosis patient.

## 2. Biochemical Properties of Vitamin A

All-trans-retinol is the molecular compound typically referred to as vitamin A. All-trans-retinol is a small, hydrophobic molecule with a cyclic ring, a polyene side chain, and a polar end group. All-trans-retinol undergoes esterification with long-chain fatty acids to produce retinyl esters (REs).

**Figure 1:**
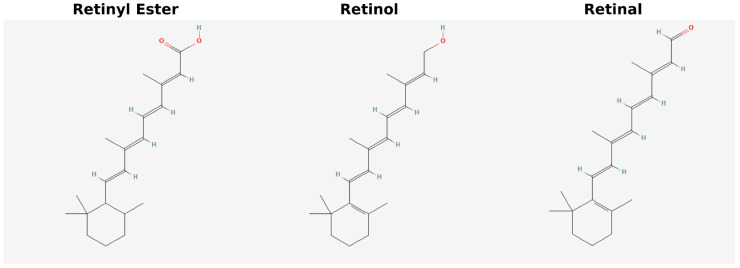
Chemical structure of common forms of vitamin A.

Retinol and REs are the principal retinoid forms present in the body. REs primarily function to store retinol and to serve as precursors in the synthesis of the visual chromophore 11-cis-retinal. The main forms of vitamin A can be seen in Figure 1. Natural variations in retinol with partial vitamin A activity also occur. For example, α-retinol, found in red palm oil and carrots, has a modified double bond and about half the bioactivity of retinol. Vitamin A_2_ (3,4-didehydroretinol) is found in freshwater fish and is also formed as a vitamin A metabolite in human skin, and it exhibits roughly 40% of retinol’s activity [1,2,3].

## 3. Vitamin A Absorption and Transport

Vitamin A absorption, which includes digestion, emulsification, intestinal uptake, intracellular metabolism, and export from the intestine into the lymphatic system or portal blood, is highly efficient in humans, with up to 90% of dietary intake absorbed within 2–6 h after digestion. REs present in foods are released via the effect of digestive enzymes in the gut. REs need to be emulsified with fatty acids and bile salts and incorporated into lipid micelles before being hydrolyzed back into retinol and fatty acids by RE hydrolases to allow for the absorption of retinol into the duodenal and jejunal enterocytes. After free retinol is taken up by enterocytes, about 95% is esterified into retinyl esters. Retinol absorption is largely unregulated, remaining efficient even at high intake levels, which can contribute to hypervitaminosis A [1,2,4].

**Figure 2:**
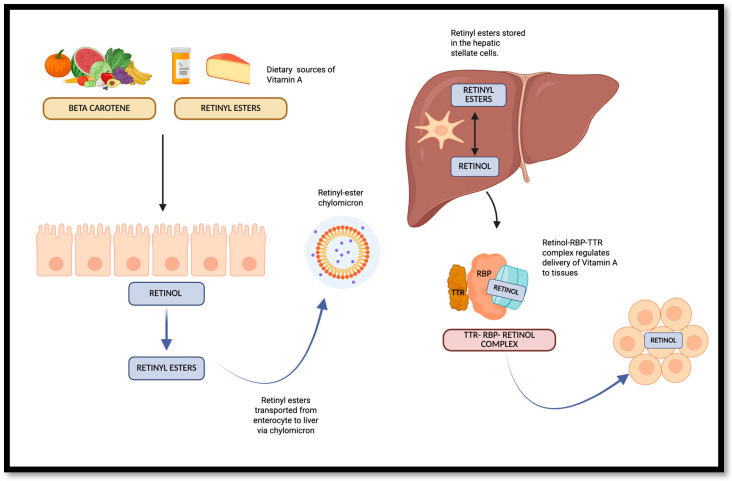
Overview of vitamin A absorption, storage and transport.

Retinol transport to tissues is a complex process that involves multiple carrier proteins (Figure 2). The two predominant pathways are those mediated by retinol bound to RBP4 in the fasting state and the postprandial delivery route. In the postprandial state, REs, together with triglycerides and cholesteryl esters, are incorporated into the lipid core of chylomicrons. The quantity of REs produced within enterocytes, as well as the amount per chylomicron particle, varies in direct proportion to the level of vitamin A absorbed and esterified at a given time, ranging from negligible to several milligrams per gram of lipid, depending on the vitamin A content of the meal. In the fasting state, retinol transport to cells primarily depends on retinol complexed with RBP4. Within the bloodstream, the retinol–RBP4 complex binds to the plasma transthyretin (TTR), which enhances the stability of the retinol–RBP4 complex and limits renal filtration. This is achieved mainly through transcellular transport of the complex by the multiligand receptor megalin found on the renal proximal tubular cells. Unbound RBP4 is freely filtered and lost in urine [1,2].

The liver is the primary site of circulating TTR production. Because TTR’s plasma concentration exceeds that of RBP4, most TTR circulates freely, with only approximately 40% bound to RBP4 in healthy humans [5]. After meals, REs contained in circulating chylomicrons and their remnants become a major source of retinoid uptake by tissues. Chylomicron triglycerides are rapidly hydrolyzed by lipoprotein lipase, leaving remnants that retain most of the original retinyl esters. Due to rapid hepatic clearance of these remnants, dietary REs exhibit a plasma half-life of less than 20 min in healthy individuals. Approximately 66–75% of chylomicron-derived retinyl esters are cleared by the liver, while peripheral tissues are responsible for the clearance of the remainder. Unlike retinol bound to RBP4, which is internalized via receptor-mediated uptake, retinoic acid is thought to mainly enter cells through passive diffusion across the phospholipid membrane without receptor involvement, though the possibility of receptor- or channel-mediated uptake has not been fully excluded. After uptake into the cell, retinoic acid is sequestered by cellular retinoic acid binding proteins, and this is thought to be a significant contributor to the higher concentration of retinoic acid in tissues as compared to serum [1,4].

In addition to facilitating postprandial retinoid delivery to the liver, hepatocytes comprise approximately 10–20% of the total hepatic retinoid storage and serve as the site of RBP4 production in the liver. Following hepatocytic uptake of postprandial retinoids, these compounds are either released back into the circulation bound to RBP4, or transferred to hepatic stellate cells for storage. In healthy individuals, roughly 70% of the body’s total retinoid reserves reside in the liver. Tissues such as the eyes, lungs, adipose tissue, skin, testes, and spleen possess the ability to store retinoids, though at considerably lower concentrations than the liver. Within adipose tissue, adipocytes serve as the primary sites of retinyl ester accumulation and are also capable of synthesizing and secreting extrahepatic RBP4. Consequently, adipocytes represent an important cell type involved in the storage and mobilization of retinoids in the body [2].

## 4. Physiologic Role of Vitamin A

Vitamin A serves two major physiological roles in ocular tissue: first, as 11-cis-retinal, it participates in photoisomerization and signal transduction within the retina, and second, as retinoic acid, it functions in the conjunctival membranes and cornea to promote epithelial cell differentiation, maintain normal morphology, and preserve barrier integrity. Vitamin A and its active metabolite, retinoic acid, function as key regulators of multiple immune cell types involved in T-cell differentiation. Retinoic acid plays a critical role in maintaining the balance between Th1 and Th2 T-helper subsets, promoting Th2 cell development. Vitamin A deficiency disrupts intestinal immune function by impairing T- and B-cell trafficking, resulting from decreased expression of adhesion molecules and lymphocyte homing receptors [1,6].

Both provitamin A carotenoids and retinoids contribute to reducing oxidative stress within the body, although they act through distinct mechanisms. Carotenoids exert direct antioxidant activity through the neutralization of reactive oxygen species, while retinoic acid functions indirectly by regulating the expression of genes such as SOD1, CAT, GPX4 and HMOX1 that are involved in the body’s endogenous antioxidant defense systems [6,7].

Retinoids also play a vital role in controlling the growth and differentiation of various skin cell types, and their deficiency results in abnormal epithelial keratinization. During tissue injury, vitamin A promotes epidermal renewal, speeds up re-epithelialization, and helps reestablish the structural integrity of the epithelium [6].

## 5. Sources of Vitamin A

The recommended dietary allowance (RDA) for vitamin A in healthy U.S. adults aged 14 years and older is 900 μg retinol activity equivalents (RAE) per day for men and 700 µg RAE per day for women. Based on 2017–2018 data from the National Health and Nutrition Examination Survey (NHANES), average daily vitamin A intake from foods and beverages in the United States was 682 μg RAE for men aged 20 years and older (approximately 76% of the RDA) and 616 μg RAE for women (approximately 88% of the RDA) [8].

The units of measurement for vitamin A are now expressed as micrograms of RAE (μg RAE), whereas International Units (IU) were previously used. RAE standardizes vitamin A activity across different forms by accounting for differences in bioavailability and conversion to retinol; for example, 1 μg RAE equals 1 μg of retinol, 2 μg of supplemental beta-carotene, or 12 μg of dietary beta-carotene. Because IU is an older activity-based unit, its conversion to μg RAE depends on the source of vitamin A; for example, 1 IU of retinol equals 0.3 μg RAE, while 1 IU of dietary beta-carotene equals 0.05 μg RAE [6].

The basal vitamin A requirement increases by a minimum of 10% during pregnancy. However, caution is advised when obtaining vitamin A from animal liver products, as excessive intake may lead to vitamin A toxicity. During pregnancy, beta-carotene (orange juice; dark green leafy vegetables) is considered the safest source for supplementation and can effectively help achieve the recommended dietary allowance (RDA) of 770 μg/day RAE. For lactating women, dietary vitamin A needs increase by about 90% to support lung maturation and immune function in the developing infant. The corresponding RDA is 1300 μg/day RAE [9].

The RDA for vitamin A was established using a stepwise approach that considered how much vitamin A the body typically loses each day via urine and feces (about 0.5% of stored amounts), along with an estimated minimum concentration in the liver of a healthy individual (around 20 micrograms per gram of liver tissue), while accounting for the average amount expected to be retained from dietary consumption. This value represents the average intake needed by the population and serves as the foundation for setting the RDA [1].

The tolerable upper intake level (UL) for vitamin A, defined as the highest daily amount unlikely to pose health risks during long-term intake, was established by the Institute of Medicine (IOM) based exclusively on preformed vitamin A. For adults, the UL is 3000 μg RAE per day, equivalent to 10,000 IU [6]. The IOM’s determination of the UL considered two primary risk factors: the risk of teratogenicity in women of reproductive age and hepatic toxicity [10].

## 6. Natural Dietary Sources (Animal vs. Plant)

The body’s requirement for vitamin A can be satisfied in two principal forms: preformed vitamin A (retinol and retinyl esters found in animal tissues) and provitamin-A carotenoids (such as β-carotene, α-carotene, and β-cryptoxanthin), mainly produced by plants. The richest food sources of preformed vitamin A are animal-based, for example, liver (especially beef), fish liver oil, and organ meats [10]. More moderate amounts are found in dairy products (milk, cheese), eggs, and fortified staples such as margarine and breakfast cereals. Table 1 highlights a few examples of foods with very high vitamin A content, according to the National Institutes of Health Office of Dietary Supplements [6].

## 7. Supplemental Forms and Bioavailability Considerations

Preformed vitamin A (retinol/retinyl esters) is highly bioavailable, with efficient intestinal absorption and direct conversion to active forms. However, excessive intake can lead to toxicity and can be teratogenic to the fetus in pregnant women. Provitamin A carotenoids—including β-carotene, α-carotene, and β-cryptoxanthin—must be converted into retinol within the intestine to become biologically active. Their absorption and utilization can vary widely depending on factors such as the food matrix, processing methods, portion size, the amount of dietary fat and fiber, and individual characteristics like gut health and genetic variation. For instance, β-carotene delivered in oil form is absorbed far more efficiently than that found in whole foods, with conversion efficiencies ranging from approximately 2:1 in oil to 12:1 or greater in a mixed diet. The primary forms of vitamin A present in dietary supplements include retinyl palmitate, which corresponds to the main retinyl ester found in animal tissues, retinyl acetate and beta-carotene. Typically, supplements will contain a combination of these molecules. These can be administered orally as capsules or liquids, or through fortified foods. Table 2 provides an overview of the forms of vitamin A and common sources [1,6,10,11,12,13].

## 8. Screening for Vitamin A Deficiency

Serum retinol concentration remains the most practical laboratory marker of vitamin A levels, with levels below 0.70 µmol/L (20 µg/dL) indicating deficiency. However, retinol is a negative acute-phase reactant and can fall transiently during systemic inflammation, limiting its utility. Serum retinol is also affected by malnutrition, and acute fluctuations may be seen when supplements or vitamin A-rich foods are consumed. Importantly, serum retinol concentration does not always accurately assess organ-level concentration of retinol. A meta-analysis by Gannon et al. found serum retinol to have 53–54% sensitivity and 79–83% specificity in reflecting reduced hepatic vitamin A levels [14].

The measurement of retinol binding protein (RBP) and its ratio to TTR is an alternate way to assess vitamin A levels. In vitamin A deficiency, RBP declines to a greater extent than TTR, leading to a lowered RBP:TTR molar ratio. During inflammation, however, both proteins decrease proportionally, so no meaningful change occurs in the RBP:TTR ratio. Emerging surrogate markers for vitamin A deficiency include the serum retinyl ester fraction (with >10% of total vitamin A suggesting excess) and the relative dose–response (RDR) test, which evaluates hepatic reserve capacity [15,16].

## 9. Vitamin A Excess and Deficiency

Vitamin A toxicity, both acute and chronic, develops when sudden or prolonged accumulation of retinoids causes functional and structural damage to multiple organ systems. Acute vitamin A toxicity is relatively uncommon but can occur when very large doses—usually exceeding 100,000 IU in adults—are ingested over a short period. Chronic vitamin A toxicity develops after months or years of intake above the tolerable upper intake level (UL) of 3000 μg RAE/day. Although acute cases can be dramatic, they usually resolve once the offending source is removed and supportive care is provided.

Clinical toxicity caused by hypervitaminosis A is uncommon in the general population; however, biochemical excess is measurable. Data from the NHANES suggest that approximately 3–6% of U.S. adults have elevated fasting retinyl esters (>10% of total vitamin A), the accepted marker for chronic hypervitaminosis A. Clinically apparent toxicity is usually iatrogenic via consumption of high doses of supplements, or excess consumption of animal organs rich in vitamin A [16].

Chronic vitamin A toxicity is far more common and begins insidiously with nonspecific symptoms such as fatigue, irritability, and anorexia before progressing to more characteristic dermatologic changes such as dry, scaly skin, cheilitis and alopecia [17]. Hepatic injury, ranging from mild transaminitis to hepatomegaly, fibrosis, or cirrhosis, and skeletal complications, such as bone pain, cortical thinning, and fragility fractures, are common. Neurologic features manifest as headaches, papilledema, and visual disturbances from elevated intracranial pressure [18]. Acute toxicity, though rare, presents within hours to days of consuming extremely high doses (>100,000 IU) with nausea, vomiting, dizziness, blurred vision, severe headache, irritability, and drowsiness, driven by sudden rises in unbound retinoids and cerebral edema; dermatologic flushing and desquamation may follow [19].

Vitamin A teratogenicity occurs with chronic excessive intake or when doses exceed 3000 μg RAE/day. The first trimester is a critical period of organogenesis, and the fetus is most susceptible to vitamin A-induced teratogenic effects during this time. Common birth defects include craniofacial abnormalities (e.g., microtia, anotia, and cleft palate) and neural tube defects. Other frequent anomalies reported include conotruncal heart defects and thymic aplasia or hypoplasia.

Accordingly, the UL for adults of both sexes was set at 3000 μg RAE per day. In contrast to retinoic acid, beta-carotene has not been shown to cause birth defects or hepatoxicity. The primary consequence of consuming excessive amounts of beta-carotene over time is carotenoderma, a harmless yellow-orange discoloration of the skin [1,5,8].

The global impact of hypovitaminosis A varies greatly across populations. The World Health Organization estimates that around 190 million preschool-aged children worldwide have low serum retinol, with the highest rates, often over 20–30%, as seen in parts of sub-Saharan Africa and South-East Asia, where deficiency remains a major driver of preventable illness and vision loss [20]. Vitamin A deficiency is rare in high-income countries and usually appears in patients with specific risk factors, especially those with fat-malabsorption disorders like cystic fibrosis, cholestatic liver disease, or inflammatory bowel disease. Even in well-resourced populations, up to 30% of adults with chronic malabsorptive conditions such as Crohn’s disease may have measurable deficiency, highlighting how vulnerable these groups remain despite adequate food availability [21]. Among older adults, national data show that the incidence of vitamin A deficiency has declined substantially over recent decades but remains a detectable problem, particularly in frail or institutionalized elderly with poor dietary intake and multiple comorbidities [22].

## 10. Intersection of Amyloid Cardiomyopathy and Vitamin A

**Figure 3:**
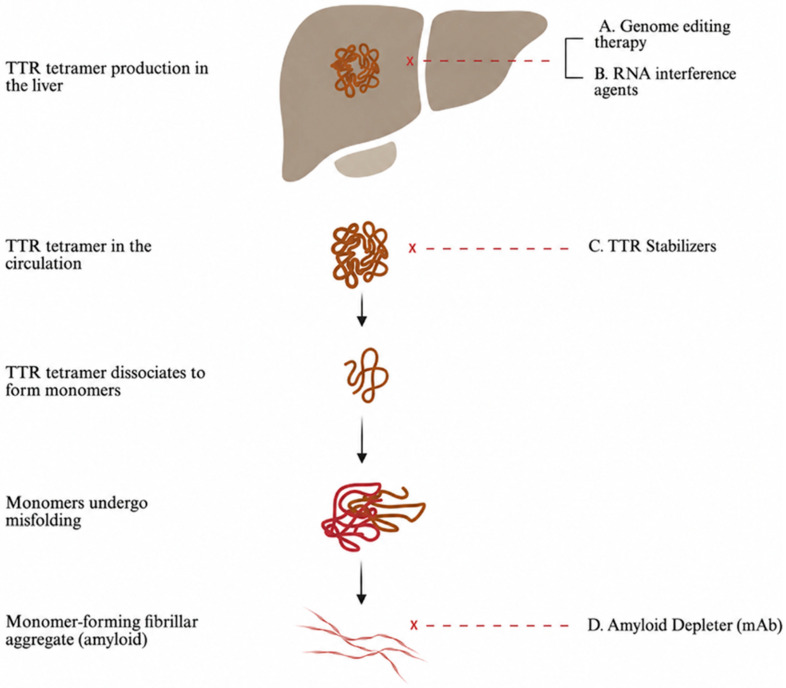
TTR amyloidogenesis and sites of action of disease-modifying therapies.

The advent of RNA-targeted therapies has significantly transformed the management of transthyretin (TTR) amyloidosis by directly reducing hepatic production of transthyretin. A schema of the therapeutic targets of commercially available therapies can be seen in Figure 3.

Patisiran is a first-generation RNA interference (RNAi) agent approved for the treatment of hereditary ATTR polyneuropathy. Administered intravenously every three weeks, it degrades TTR mRNA in hepatocytes, thereby lowering the circulating TTR levels. In the landmark APOLLO trial, patisiran significantly improved neuropathy impairment scores and quality-of-life measures compared with placebo. Vutrisiran is a second-generation RNAi agent, approved for both ATTR polyneuropathy and ATTR cardiomyopathy. It is administered as a subcutaneous injection every three months, offering patient convenience while maintaining sustained TTR suppression. Eplontersen is an antisense oligonucleotide approved for ATTR polyneuropathy that directly binds TTR mRNA, resulting in its degradation and reduced hepatic TTR synthesis. It is administered as a once-monthly subcutaneous injection. Across studies, all three agents reduce circulating TTR levels by about 81–87% and slow disease progression in amyloidosis [23,24,25].

CRISPR Cas9 gene-editing therapy is under phase III investigation to treat hereditary amyloidosis by reducing transthyretin (TTR) production by up to 97%. A potential advantage of CRISPR-based therapy is that it does not require frequent dosing. Because CRISPR significantly decreases TTR production in hepatocytes, patients treated with this approach receive vitamin A supplementation as a precautionary measure against possible vitamin A deficiency [26]. Monoclonal antibodies such as ALXN2220 (N1006) and coramitug (PRX004) are also under phase-III investigation for both hereditary and variant ATTR-CM. Antibody therapies work by binding to pathologic TTR fibrils and stimulating immune clearance of the antibody-TTR complex. It would not be expected that these therapies would influence vitamin A levels [27].

Concurrent vitamin A supplementation is recommended for patients receiving TTR-silencing therapies. Current recommendations advise that all patients on TTR-silencing therapies receive the daily recommended allowance of vitamin A to reduce the risk of deficiency [28,29,30].

Various clinical trials of TTR-silencing therapies have documented reductions in circulating vitamin A levels and monitored for potential deficiency in the treatment arms. In contrast to TTR stabilizers, such as tafamidis or acoramidis—which preserve circulating TTR levels by stabilizing the tetramer and do not affect vitamin A status—TTR gene-silencing therapies act by reducing hepatic TTR production, resulting in lower circulating TTR levels and corresponding decreases in serum vitamin A. In the APOLLO trial, 99% of patients with normal baseline vitamin A levels developed low serum vitamin A during treatment with patisiran. Despite this, only one case was reported as an adverse reaction, and no clear clinical manifestations of vitamin A deficiency were observed [31]. In the HELIOS-A and HELIOS-B trials, all patients in the vutrisiran treatment arms were instructed to take the recommended daily allowance of vitamin A throughout the study to offset the anticipated reduction in serum vitamin A levels.

In HELIOS-A, 98% of patients with normal baseline serum vitamin A levels developed low circulating vitamin A, and HELIOS-B saw 80% of patients with normal baseline vitamin A levels developing low serum levels [28]. Despite these reductions, pooled safety analyses from both trials did not show evidence of ocular adverse events attributed to vitamin A deficiency [30]. In the NEURO-TTRansform trial, circulating vitamin A deficiency was among the most frequently reported adverse events in the eplontersen group. However, the proportion of ocular events was similar in the eplontersen group (17%) and the placebo group (15%) [23].

Collectively, these trials show that TTR-silencing therapies consistently lower serum vitamin A levels. However, none have provided convincing clinical evidence of symptomatic vitamin A deficiency. Although vitamin A status was assessed, these studies were not designed for long-term evaluation, and it remains possible that extended follow-up could reveal a higher incidence of clinically apparent deficiency. Within the duration of the available studies, however, clear evidence of symptomatic deficiency has not been observed. One possible explanation is that although circulating vitamin A decreases, hepatic stores remain largely preserved. The liver is the primary storage organ for vitamin A, and TTR-silencing therapies appear to reduce circulating levels without significantly depleting hepatic reserves. Another possible explanation is the presence of alternative transport mechanisms. Knockout mouse models of TTR and RBP4 have been shown to be compatible with life. Although these models demonstrate markedly reduced plasma retinol and RBP4 levels, hepatic and peripheral tissue retinol and retinyl ester concentrations remain normal, and the animals do not develop clinical features of vitamin A deficiency. This may be due to TTR-independent uptake of vitamin A from the gastrointestinal tract into the liver. In addition, albumin, thyroid-binding globulin, and lipoproteins have been proposed as potential surrogate carriers of RBP4 in TTR-deficient states [32].

## 11. Conclusions

TTR amyloid cardiomyopathy is a systemic, progressive and potentially fatal disease if left untreated. There has been an expansion in available therapeutic options, such as RNAi and, potentially, CRISPR-based therapies, which target a reduction in circulating TTR. TTR plays an integral role in vitamin A transport through its stabilization of RBP4, and as such, a decrease in circulating TTR can lead to a reduction in circulating vitamin A levels. Though all published clinical trials with TTR-directed RNAi therapies have demonstrated decreases in circulating vitamin A levels, clinically meaningful events related to vitamin A deficiency have not been demonstrated. Alternate, RBP4-independent pathways of vitamin A transport may mitigate potential clinical vitamin A deficiency. The current recommendation for all patients on TTR-silencing therapies is to take vitamin A supplementation. The long-term effect of TTR suppression on clinical vitamin A deficiency, though encouraging, is currently the subject of post-marketing surveillance and further research.

## Figures and Tables

**Table 1:** Foods rich in vitamin A and their approximate vitamin A contents.

Food Source	Approximate Vitamin A Contents (%DV)
Beef liver, pan-fried (3 oz)	6582 µg RAE (731)
Sweet potato, baked in skin (1 whole)	1403 µg RAE (156)
Spinach (½ cup)	573 µg RAE (64)
Pumpkin pie, commercially prepared (1 piece)	488 µg RAE (54)
Carrots, raw (½ cup)	459 µg RAE (51)

Adapted from Office of Dietary Supplements (ODS), National Institutes of Health (NIH). Vitamin A and Carotenoids—Fact Sheet for Health Professionals [6].

**Table 2:** Summary of vitamin A forms and common sources.

Form of Vitamin A	Type	Description	Food Sources
Retinol (Vitamin A by definition)	Preformed vitamin A (Directly bioavailable; used by the body without conversion)	Predominant form in the human body. Retinol is essential for the synthesis of retinal and retinoic acid and has direct biological functions in cellular metabolism and stem cell maintenance.	Animal-derived foods are the main dietary sources of preformed vitamin A, primarily in the form of retinyl esters.
Retinyl Esters (RE)		After ingestion, they’re hydrolyzed to retinol, absorbed, re-esterified, and transported to the liver for storage.	
Retinal (Retinaldehyde)	Metabolites of preformed vitamin A	11-cis-retinal binds to opsin proteins to form rhodopsin, the light-sensitive pigment essential for vision.	Not found preformed in the diet.
Retinoic Acid (RA)		RA regulates gene expression, cell growth, and differentiation through RAR/RXR receptors.	
β-Carotene	Provitamin A	Beta-carotene is the most common provitamin A carotenoid; however, alpha-carotene and beta-cryptoxanthin exhibit higher bioavailability from dietary sources.	Humans cannot synthesize carotenoids. The major sources of carotenoids in the human diet are deeply pigmented yellow to red fruits and vegetables.
α-Carotene			
β-Cryptoxanthin			

## Data Availability

No new data were created or analyzed in this study. Data sharing is not applicable to this article.

## Abbreviations

The following abbreviations are used in this manuscript:

TTR	Transthyretin
RBP4	Retinol-binding protein
ATTR-CM	TTR amyloid cardiomyopathy
RE	Retinyl ester
RDA	Recommended daily allowance
RAE	Retinol activity equivalents
NHANES	National Health and Nutrition Examination Survey
UL	Upper intake level
